# The price of pressure: nationwide survey on lifestyle disturbances, occupational burnout and compromised perceived-competency among radiology residents in China

**DOI:** 10.3389/fpubh.2024.1472397

**Published:** 2024-10-23

**Authors:** Zeqi Liu, Qinqi Yao, Peicheng Wang, Lijun Shen, Hange Li, Jingfeng Zhang, Maoqing Jiang, Zhenghan Yang, Zhenchang Wang, Jianjun Zheng, Jiming Zhu, You Wu

**Affiliations:** ^1^Institute for Hospital Management, Tsinghua University, Beijing, China; ^2^Vanke School of Public Health, Tsinghua University, Beijing, China; ^3^Department of Radiology, Hwa Mei Hospital, University of Chinese Academy of Sciences, Ningbo, China; ^4^Department of Radiology, Beijing Friendship Hospital, Capital Medical University, Beijing, China; ^5^Vanke School of Public Health, Institute for Healthy China, Tsinghua University, Beijing, China; ^6^Institute for Hospital Management, School of Basic Medical Sciences, Tsinghua Medicine, Tsinghua University, Beijing, China

**Keywords:** radiology residents, lifestyle factors, perceived-competency, burnout, China

## Abstract

**Objectives:**

The competency of radiology directly affects the quality and equity of medical services. Due to their different occupational characteristics compared to other specialists, this study aims to evaluate the impacts of lifestyles on competency and burnout in radiology residents in China.

**Materials and methods:**

A nationwide, cross-sectional survey was conducted from December 1, 2020 to April 30, 2021. A total of 12,208 radiology residents during their standardized residency training in China were invited. Multivariate linear regression and logistic regression were conducted to identify perceived competency and burnout associated with lifestyles.

**Results:**

Of the 3,666 participants, 58.02% were female, 82.24% were aged <30 years, 40.53% were from the Eastern region, and 92.06% obtained a bachelor’s degree. The radiology residents with high-level lifestyles had higher competency (*β =* 0.16, 95% CI = [0.01, 0.32]), particularly in the realms of sleep, physical activity, and alcohol consumption. The correlation was stronger in residents with longer work hours and more night shifts. Residents with insomnia (OR = 7.69, 95% CI = [4.23, 14.67]) and less exercise (OR = 6.24, 95% CI = [1.33, 29.37]) had higher burnout risk, while residents who smoked had a lower risk (OR = 0.60, 95% CI = [0.40, 0.89]). And lifestyle factors had a slightly different impact on emotional exhaustion and depersonalization.

**Conclusion:**

Radiology residents’ lifestyles can be emphasized, as it may reflect their pressure and wellbeing and influence their concentration, competency, burnout and performance. Policymakers and hospital administrators should incorporate practical and modifiable strategies into work routines to improve the lifestyle quality of residents.

## Introduction

Radiologists play an important role in health care delivery, directly determining the quality (1) and accessibility of medical services. Competence is the use of one’s acquired clinical skills, knowledge, communication and values to provide professional services (2). Assessing competence traditionally involves objective metrics but increasingly acknowledges the value of self-perceived competency assessments. Despite their subjective nature, these assessments provide crucial insights into how individuals perceive their own capabilities, which can significantly influence professional development and performance. Spännargård et al. (3) found significant associations between perceived clinical competence, workplace setting, and varying levels of burnout among psychotherapists, underscoring the importance of self-perceived competence assessments in professional wellbeing (3).

Previous research has suggested the impacts of training, increased experience (4, 5), overnight assignments (6), dissatisfaction with the work-life balance (7), and satisfaction with training (8) on clinical competencies of medical professionals. The quality of life at work for healthcare workers are another potential influencing factor (9), yet, owing to their occupational characteristics, radiologists were considered one of the high-risk groups with poor quality of life (10). Evidence has shown that the mouse activity of radiology residents reached up to a distance of 2.2 kilometers and 23 keystrokes per minute (11), and that most radiology residents spend at least 6 h per workday seated—a duration exceeding that of residents from other specialties (12). However, the association between lifestyle and competency among radiologists remains unclear. The quality differences in the cross-sectional imaging modality, including computed tomography and ultrasound, increased during the final 2 h of continuous overnight shifts of radiology residents (13).

Burnout is another prime factor inhibiting performance. Evidence has shown that doctors with high levels of occupational burnout are more likely to make medical errors and have poorer health, psychological and career outcomes (14). A recent systematic review and meta-analysis showed that about half of the radiology residents showed at least one of the three burnout manifestations, with a moderate to high degree of severity (15). For a long time, lifestyle has been able to determine the psychological agenda. It has been reported work performance reduces with increased burnout which is aggravated due to poor sleep quality (16). As a psychological syndrome, occupational burnout is deeply affected by the lifestyle of radiologists. Romani concluded that more exercises can reduce anxiety levels and exhaustion symptoms while improving the mental and physical wellbeing of healthcare workers (15). It was reported that the lack of exercise was one of the predictors of radiology residents’ burnout, which further affects their performance (OR = 0.31, 95% CI = 0.10–1.00) (7). However, the current research on the lifestyle and occupational burnout of radiology residents is insufficient, and more evidence is needed.

Given the potential for radiology residents in standardized residency training to significantly improving their professional skills, our study focuses on the impacts of lifestyle behaviors on perceived-competency and burnout among radiology residents in China, using data from a nationwide survey. This study aims to contribute to improving the work conditions of radiologists and the associated quality of care, which is significant for both the doctors themselves and the health care system.

## Materials and methods

### Study design and participants

The Institutional Review Board approval and subject informed consent were obtained before the study began [Tsinghua University, China (No. 20210140)]. This prospective survey study was conducted by the Chinese Association of Radiologists (CAR), between December 1, 2020 and April 30, 2021. The survey population comprised radiology residents during their standardized residency training across 31 provinces (data on Macao, Hong Kong and Taiwan are not available) in China. Data were collected using a self-administered and structured questionnaire, which was an anonymous web-based survey issued via a popular online survey platform called “Wenjuanxing” in China. At the start of the online link, consent was required to continue, and the respondents could withdraw their information at any time. A total of 3,666 out of 12,208 potentially eligible radiologists who were receiving standardized residency training during 2020 in China responded effectively to our survey. They came from 97 cities in 31 provinces of China, and 407 (73.1%) of 557 radiology programs were covered.

### Exposure and outcome measurements of radiology residents

#### Lifestyles factors

In terms of lifestyles, as shown in Table 1, the radiology residents were asked a series of four questions assessing the quality of their sleep (normal, occasional insomnia, sometimes insomnia, frequent insomnia and almost daily insomnia), exercise (none, occasionally, sometimes, often, almost every day), smoking (non-smoker, smoker) and drinking habits (never, 1 time or less per month, 2–4 times per month, 2–3 times per week, 4 or more times per week). Each question was granted a five-point scale ranked from healthy state to unhealthy state, except smoking as a binary variable.

**Table 1:** Definition of lifestyle factors.

Lifestyle factors	Definition
Sleep	Almost daily insomnia = 0, Frequent insomnia = 0.25, Sometimes insomnia = 0.5, Occasional insomnia = 0.75, Normal = 1
Physical activities	None = 0, Occasionally = 0.25, Sometimes = 0.5, Often = 0.75, Almost every day = 1
Smoking	Smoker = 0, Non-smoker = 1
Drinking habits	4 or more times per week = 0, 2–3 times per week = 0.25, 2–4 times per month = 0.5, 1 time or less per month = 0.75, Never = 1
Overall scores	Low = (≤ 2.5), Moderate = (2.5–3), High (= >3)

The scores of sleep, physical activities and alcohol consumption were converted to the scale of 0 to 1 point. The overall healthy lifestyle scores were categorized into low (≤ 2.5), moderate (2.5–3) and high (>3) levels to reflect the least healthy lifestyle, the moderate lifestyle, and the healthiest lifestyle, respectively.

#### Perceived-competency

The Diagnostic Radiology Milestones were designed to evaluate residents in the context of their participation in ACGME (The Accreditation Council for Graduate Medical Education)-accredited residency programs (17). We developed the questionnaire part by adopting the conceptual framework of the ACGME Six Core Competencies and selecting 9 detailed milestones from 24 ones out of 6 competency domains. Milestones are arranged into levels, as shown in Table 2, and tracking from Level 1 to Level 5 implies from the novice to the expert resident, and Level 4 is designed as a graduation goal but does not represent a graduation requirement. The five levels were evaluated on a 9-point scale, as described in our previous research (8). And the total score was calculated based on the average score of 6 domains. Participants scoring ≥4 were considered as a high level of perceived-competency.

**Table 2:** Definition of perceived-competency.

Perceived-competency	Definition
Patient care	1 = level 1, 2 = between level 1 and level 2, 3 = level 2, 4 = between level 2 and level 3, 5 = level 3, 6 = between level 3 and level 4, 7 = level 4, 8 = between level 4 and level 5, 9 = level 5
Medical knowledge	
Systems-based practice	
Practice-based learning and improvement	
Professionalism	
Interpersonal and communication skills	
Overall mean scores	High level = (≥4)

#### Occupational burnout

Occupational burnout was measured by the MBI-HSS (Maslach Burnout Inventory – Human Service Survey) questionnaire, a 22-item, 7-point Likert-type scale self-report measure instrument, which consists of three dimensions of emotional exhaustion (EE), depersonalization (DP) and personal accomplishment (PA). We adopted the dimensions of emotional exhaustion and depersonalization, containing nine items and five items, and reduced the personal accomplishment part (18, 19). High scores correspond to high feelings of burnout. Occupational burnout was defined as emotional exhaustion scores ≥27 or depersonalization scores ≥10 (20, 21). In the dimensions of emotional exhaustion, total scores range from 0 to 16 were considered a low level of EE, range from 17 to 26 were considered moderate, and scores ≥27 were considered high. In the dimensions of depersonalization, total scores range from 0 to 6 were considered a low level of EE, range from 7 to 12 were considered moderate, and scores ≥13 were considered high (22).

#### Socio-demographic characteristics

Socio-demographic variables include gender (male and female), age (≤25 years, 25–30 years, and > 30 years), region (East, Central Region, West, and North West), degree (bachelor’s degree, master’s and doctoral degree), marital status (unmarried and married), children (have children and no child), work years (≤3 and > 3 years), work hours per day (≤8 and > 8 h), number of night shifts (≤1 time/month, 2–3 times/month, 1–2 times/week, and ≥ 3 times/week), residency training year (first year, second year, and third year) and residency training site (general tertiary A, specialist tertiary A, and other sites).

### Statistical analysis

Descriptive analysis was calculated for continuous variables [mean, standard deviation (SD)] and categorical variables (*n*, %). The percentages of lifestyles among residents, distribution of competency-based performance and prevalence of burnout with confidence interval were calculated. Pearson’s chi-square test was performed to test associations between socio-demographics and lifestyle behaviors, perceived-competency and occupational burnout. Spearman’s correlation analysis was carried out among the lifestyle factors.

Multiple linear regression models were constructed to explore the relationship of lifestyles and perceived-competency, adjusted for socio-demographics. To further examine the impact of different lifestyle factors on competency, we constructed multivariable logistic regression models to obtain the odds ratios (ORs) and 95% confidence intervals (CIs) comparing lower and higher competency. Besides, to detect any discrepancy in the relationship between lifestyles and competency among residents with different characteristics, subgroup analyses were performed according to socio-demographic variables. To explore the relationship between lifestyles and occupational burnout, logistic regression models were used to identify predictive lifestyle factors to occupational burnout and each subscale (EE, DP). The association was presented as odds ratios (OR) and 95% confidence intervals (CIs).

Two-tailed *p* < 0.05 were considered statistically significant. Statistical analyses were performed using Stata 17.0 (Stata Corporation) and visualized using R 4.2.3 (R Foundation for Statistical Computing) for Windows.

## Results

### Socio-demographics factors

A summary of the socio-demographic characteristics of the radiology residents is shown in Table 3. Of the 3,666 participants, most were female (58.02%), aged 25–30 (64.62%), and had a balanced regional distribution with fair representation from the Eastern (40.53%), Central (20.24%), and Western (33.28%) regions. Most held a bachelor’s degree (92.06%), were unmarried (77.63%), and childless (86.93%). Work experience was generally under 3 years (77.66%), with a majority working 8 h daily (72.15%) and not frequently on night duty (61.65%). Residents were evenly distributed across training years, with 96.84% trained in general tertiary A hospitals. Competency varied by several factors, while burnout was influenced by gender, location, family structure, and work patterns.

**Table 3:** Summary of socio-demographic characteristics of the radiology residents.

	*n* (%)	Lifestyle		Perceived-competency		Burnout	
		*χ* ^2^	*p*-value	*χ* ^2^	*p*-value	*χ* ^2^	*p*-value
Gender		81.55	**<0.001**	17.68	**<0.001**	57.70	**<0.001**
Male	1,539 (41.98%)						
Female	2,127 (58.02%)						
Age		18.50	**0.001**	79.90	**<0.001**	0.38	0.828
≤25	895 (24.41%)						
25<x ≤ 30	2,369 (64.62%)						
>30	402 (10.97%)						
Location		23.17	**0.001**	6.89	0.076	18.71	**<0.001**
East	1,486 (40.53%)						
Central region	742 (20.24%)						
West	1,220 (33.28%)						
North West	218 (5.95%)						
Degree		10.76	**0.029**	11.22	**0.004**	3.71	0.156
Bachelor	3,375 (92.06%)						
Master	229 (6.25%)						
Doctoral	62 (1.69%)						
Marital status		23.98	**<0.001**	22.75	**<0.001**	0.00	0.973
Married	820 (22.37%)						
Unmarried	2,846 (77.63%)						
Children		18.87	**<0.001**	10.48	**0.001**	4.31	**0.038**
No	3,187 (86.93%)						
Yes	479 (13.07%)						
Work years		41.18	**<0.001**	29.32	**<0.001**	3.94	**0.047**
≤3	2,847 (77.66%)						
>3	819 (22.34%)						
Work hours per day		112.33	**<0.001**	1.26	0.261	77.95	**<0.001**
≤8	2,645 (72.15%)						
>8	1,021 (27.85%)						
Number of night shifts		72.68	**<0.001**	38.42	**<0.001**	29.96	**<0.001**
Once a month or none	2,260 (61.65%)						
2–3 times a month	617 (16.83%)						
1–2 times every week	635 (17.32%)						
≥3 times every week	154 (4.20%)						
Residency training year		33.95	**<0.001**	164.69	**<0.001**	5.03	0.081
First year	1,285 (35.05%)						
Second year	1,179 (32.16%)						
Third year	1,202 (32.79%)						
Residency training site tier		3.60	0.463	0.87	0.648	4.69	0.096
General tertiary A	3,550 (96.84%)						
Specialist tertiary A	80 (2.18%)						
Other	36 (0.98%)						

The bold values indicate significant results.

### Participants’ lifestyle, perceived-competency and burnout prevalence

As shown in Table 4, the overall mean score for lifestyle behaviors was 2.79 (of 4), which indicates a moderately healthy lifestyle. Among the 4 scales, sleep, smoking, and alcohol consumption showed healthy behaviors of radiology residents, while they lacked physical activities. For perceived-competency, the overall mean score was 3.16 (of 9), indicating a low competence level, with the highest score reported in interpersonal and communication skills (3.51). Prevalence of occupational burnout in all participants was 25.78%, the proportions of radiology residents who scored high level in EE and DP were 16.83 and 23.19%, respectively.

**Table 4:** Participants’ lifestyle and perceived-competency and burnout (*N* = 3,666).

Variables	*n*/Mean	%/SD
Lifestyle		
Sleep	Mean score (SD):	0.76 (0.26)
Normal	1,608	43.86%
Insomnia< twice a month	1,028	28.04%
Insomnia 1–2 times a week	653	17.81%
Insomnia 3–5 times a week	324	8.84%
Insomnia almost every day	53	1.45%
Physical activity	Mean score (SD):	0.22 (0.22)
Never	1,374	37.48%
< twice a month	1,518	41.41%
1–2 times a week	584	15.93%
3–5 times a week	167	4.56%
Almost every day	23	0.63%
Smoke	Mean score (SD):	0.96 (0.20)
No	3,516	95.91%
Yes	150	4.09%
Alcohol consumption	Mean score (SD):	0.85 (0.16)
Never	1,695	46.24%
≤once a month	1721	46.94%
2–4 times a month	218	5.95%
2–3 times a week	27	0.74%
≥4 times a week	5	0.14%
Overall mean score (0–4)	2.79	0.47
Perceived-competency		
Patient care	2.85	1.45
Medical knowledge	3.36	1.63
Systems-based practice	2.96	1.68
Practice-based learning and improvement	3.01	1.79
Professionalism	3.28	1.87
Interpersonal and communication skills	3.51	2.07
Overall mean score (0–9)	3.16	1.50
Burnout		
Emotional exhaustion		
Low	2,371	64.68%
Moderate	678	18.49%
High	617	16.83%
Depersonalization		
Low	1,962	53.52%
Moderate	854	23.30%
High	850	23.19%
Overall prevalence	945	25.78%

### Lifestyle factors and perceived-competency

The correlations among sleep, physical activity, smoking, and alcohol assumption were low (Supplementary Table 1), suggesting that there was a slight impact of confounders or collinearity. After adjusted for sociodemographic covariates (Supplementary Table 2 and Table 5), the radiology residents with high-level lifestyles had higher perceived-competency (*β =* 0.22, 95% CI = [0.08, 0.35]) compared with lifestyle behaviors scoring low, especially in medical knowledge (*β =* 0.16, 95% CI = [0.01, 0.30]), systems-based practice (*β =* 0.22, 95% CI = [0.07, 0.37]), practice-based learning and improvement (*β =* 0.24, 95% CI = [0.08, 0.40]), professionalism (*β =* 0.30, 95% CI = [0.13, 0.47]), and interpersonal and communication skills (*β =* 0.29, 95% CI = [0.10, 0.48]). Also, sleep was marginally associated with competency (*β =* 0.14, 95% CI = [0.02, 0.26]; *β =* 0.20, 95% CI = [0.09, 0.32]). Compared with residents who seldom exercise, those who had regular physical activities had higher competence (*β =* 0.25, 95% CI = [0.14, 0.37]). Among residents who consume alcohol, those consuming moderate and low alcohol had lower competence than those consuming high alcohol (*β = −*0.26, 95% CI = [−0.45, −0.07]; *β = −*0.35, 95% CI = [−0.58, −0.17]). Nevertheless, there was no significant association between smoke and competency-based performance among radiology residents.

**Table 5:** Differences and 95% CI of perceived-competency according to lifestyle scores of radiology residents.^1^

Variables	Competency-based performance															
	Patient care		Medical knowledge		Systems-based practice		Practice-based learning and improvement		Professionalism		Interpersonal and communication skills		Overall performance		Overall high performance^6^	
	*β*	95% CI	*β*	95% CI	*β*	95% CI	*β*	95% CI	*β*	95% CI	*β*	95% CI	*β*	95% CI	OR	95% CI
Overall healthy lifestyle^2^																
Low	Ref		Ref		Ref		Ref		Ref		Ref		Ref		Ref	
Medium	0.04	(−0.06, 0.15)	0.11	(−0.01, 0.23)	0.05	(−0.07, 0.18)	0.12	(−0.02, 0.25)	**0.14**	**(0.00, 0.28)**	**0.16**	**(0.00, 0.32)**	0.10	(−0.01, 0.21)	1.08	(0.91, 1.30)
High	0.09	(−0.04, 0.22)	**0.16**	**(0.01, 0.30)**	**0.22**	**(0.07, 0.37)**	**0.24**	**(0.08, 0.40)**	**0.30**	**(0.13, 0.47)**	**0.29**	**(0.10, 0.48)**	**0.22**	**(0.08, 0.35)**	1.21	(0.98, 1.50)
Sleep^3^																
Insomnia	Ref		Ref		Ref		Ref		Ref		Ref		Ref		Ref	
Almost normal	0.09	(−0.03, 0.21)	**0.14**	**(0.01, 0.27)**	**0.15**	**(0.01, 0.29)**	0.11	(−0.04, 0.26)	0.14	(−0.02, 0.30)	**0.21**	**(0.04, 0.39)**	**0.14**	**(0.02, 0.26)**	1.13	(0.92, 1.38)
Normal	0.08	(−0.03, 0.19)	**0.17**	**(0.05, 0.29)**	**0.21**	**(0.08, 0.34)**	**0.20**	**(0.07, 0.34)**	**0.24**	**(0.10, 0.39)**	**0.30**	**(0.14, 0.47)**	**0.20**	**(0.09, 0.32)**	**1.24**	**(1.03, 1.50)**
Physical activity^4^																
Irregular	Ref		Ref		Ref		Ref		Ref		Ref		Ref		Ref	
Regular	**0.17**	**(0.06, 0.28)**	**0.22**	**(0.09, 0.34)**	**0.27**	**(0.14, 0.40)**	**0.25**	**(0.11, 0.39)**	**0.32**	**(0.18, 0.47)**	**0.28**	**(0.12, 0.44)**	**0.25**	**(0.14, 0.37)**	**1.40**	**(1.17, 1.67)**
Smoking																
Yes	Ref		Ref		Ref		Ref		Ref		Ref		Ref		Ref	
No	−0.19	(−0.42, 0.05)	−0.10	(−0.36, 0.16)	−0.15	(−0.42, 0.12)	0.02	(−0.28, 0.31)	−0.14	(−0.45, 0.17)	−0.16	(−0.51, 0.18)	−0.12	(−0.36, 0.12)	0.77	(0.54, 1.10)
Alcohol consumption^5^																
High	Ref		Ref		Ref		Ref		Ref		Ref		Ref		Ref	
Moderate	**−0.28**	**(−0.46, −0.09)**	**−0.22**	**(−0.43, −0.01)**	**−0.26**	**(−0.48, −0.04)**	**−0.28**	**(−0.51, −0.05)**	−0.21	(−0.46, 0.03)	**−0.30**	**(−0.57, −0.03)**	**−0.26**	**(−0.45, −0.07)**	**0.64**	**(0.48, 0.85)**
Low	**−0.32**	**(−0.51, −0.12)**	**−0.32**	**(−0.54, −0.10)**	**−0.35**	**(−0.58, −0.12)**	**−0.38**	**(−0.63, −0.14)**	**−0.38**	**(−0.64, −0.12)**	**−0.49**	**(−0.78, −0.20)**	**−0.37**	**(−0.58, −0.17)**	**0.53**	**(0.39, 0.72)**

^1^Generalized linear models adjusted for gender (male, female), age (≤25 years, 25 years < x ≤ 30 years, >30 years), region (east, central region, west, north east), degree (bachelor, master, doctoral), marital status (married, unmarried), children (yes, no), work years ( ≤ 3 years, > 3 years), work hours per day ( ≤ 8 h, > 8 h), number of night shifts ( ≤ 1 time/month, 2–3 times/month, 1–2 times/week, ≥ 3 times/week), residency training year (1st year, 2nd year, 3rd year) and residency training site (general tertiary A, specialist tertiary A, other). ^2^Total scores of no more than 2.5 was considered a low level of healthy lifestyle behaviors, scores from 2.5 to 3 were considered medium, and scores above 3 were considered high. ^3^Unable to sleep at least 1–2 times a week was considered insomnia, and insomnia greater than twice a month was considered basically normal. ^4^Exercise less than twice a month was considered irregular, exercise at least once a week was considered regular. ^5^None of alcohol assumption was considered a low level, alcohol assumption less than once a month was considered moderate, and drinking more than twice a month was considered high. ^6^Total scores of no more than 4 was considered a low level of competency-based performance, and scores above 4 were considered high. The bold values indicate significant results.

As shown in Table 5, after transforming the perceived-competency into the categorical variable, it appears that the relationship between lifestyle behaviors and overall high performance was not noticeable (OR = 1.08, 95% CI = [0.91, 1.30]; OR = 1.21, 95% CI = [0.98, 1.50]). However, residents with normal sleep were 1.24 times (95% CI = [1.03, 1.50]) more likely to develop high performance than those who had insomnia, and residents with regular physical activities were 1.40 (95% CI = [1.17, 1.67]) times than residents who exercise infrequently. Residents who consumed moderate and low alcohol were 0.64 (95% CI = [0.48, 0.85]) and 0.53 (95% CI = [0.39, 0.72]) times, respectively, than those who drank more.

**Figure 1:**
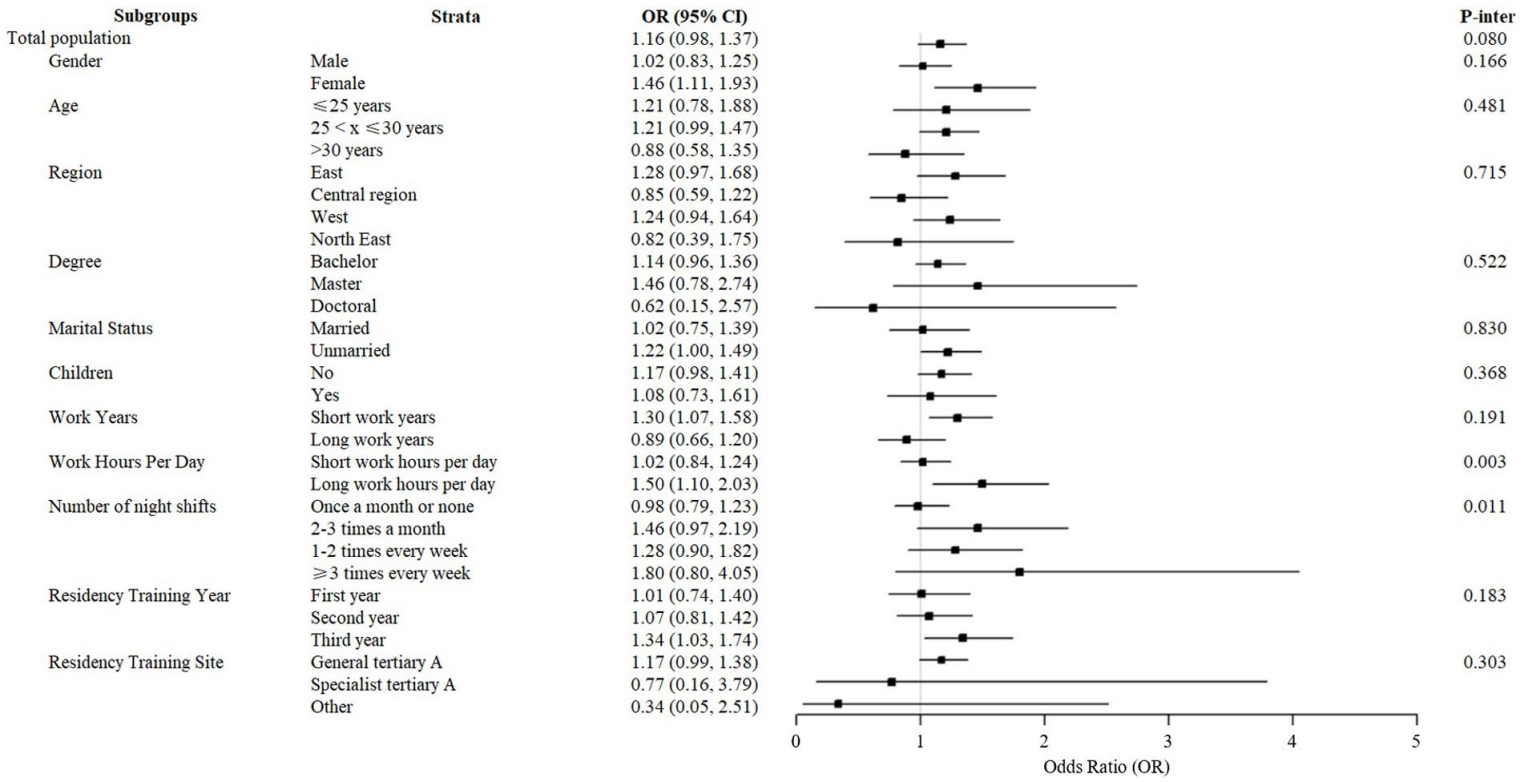
Subgroup analyses of the relationship of lifestyle and perceived-competency by gender, degree, region, marital status, work hours per day, number of night shifts, and residency training site.

In subgroup analyses, there were obvious differences in the correlation between lifestyle and perceived-competency in terms of work hours per day and night shifts (Figure 1 and Supplementary Table 3). These associations were stronger among residents working for over 8 h per day (*p* = 0.003) and rotating more night shifts (*p* = 0.011). Specifically, as shown in Supplementary Table 4, radiology residents with more work hours per day and more night shifts were more likely to suffer from sleep disorders (*p* < 0.001; *p* < 0.001), and had fewer physical activities (*p* < 0.001; *p* = 0.009), likewise, radiology residents with more night shifts were more likely to drink more alcohol (*p* < 0.001), which led to a closer association between lifestyle and competency.

### Lifestyle factors and occupational burnout

Table 6 reveals the results of the analysis of lifestyle factors associated with occupational burnout by a multivariate logistic regression. Smoking, sleep, and physical activity were independently associated with occupational burnout, while the effect of alcohol consumption was non-significant. The OR of smoking, alcohol consumption, sleep and physical activity factors were 0.60 (95% CI = [0.40, 0.89], 1.23) (95% CI = [0.09, 17.59]), 7.69 (95% CI = [4.23, 14.67]), and 6.24 (95% CI = [1.33, 29.37]), respectively, comparing the worst to the healthiest state. This demonstrated that participants who suffer insomnia almost every day and never exercise had higher risk for occupational burnout when compared with those with healthy lifestyles, while participants who smoke show the lower risk.

**Table 6:** Logistic regression model of burnout and their lifestyle factors.

Variables	Odds ratio	95% CI	*p*-value
Smoking			
No			
Yes	**0.60**	**(0.40, 0.89)**	**0.013**
Alcohol consumption			
Never			
≤ once a month	1.11	(0.92, 1.33)	0.293
2–4 times a month	1.12	(0.79, 1.60)	0.524
2–3 times a week	1.42	(0.64, 3.16)	0.387
≥4 times a week	1.23	(0.09, 17.59)	0.881
Sleep			
Normal			
Insomnia < twice a month	**1.65**	**(1.34, 2.02)**	**<0.001**
Insomnia 1–2 times a week	**2.82**	**(2.26, 3.52)**	**<0.001**
Insomnia 3–5 times a week	**5.73**	**(4.36, 7.54)**	**<0.001**
Insomnia almost every day	**7.96**	**(4.32, 14.67)**	**<0.001**
Physical activity			
Almost every day			
3–5 times a week	4.70	(0.97, 22.87)	0.055
1–2 times a week	3.29	(0.69, 15.62)	0.134
< twice a month	3.68	(0.78, 17.34)	0.099
Never	**6.24**	**(1.33, 29.37)**	**0.021**

The bold values indicate significant results.

Secondary analysis was conducted similarly for emotional exhaustion (EE) and depersonalization (DP) subscale to identify the specific association between lifestyles and occupational burnout. As shown in Supplementary Table 5, 6, the logistic regression analysis revealed that high EE was predicted by sleep (insomnia almost every day versus never; OR = 15.08, 95% CI = [7.16, 31.73]) and physical activity (never exercise versus almost every day, OR = 10.06; 95% CI = [1.23, 82.51]), high DP was predicted by sleep (insomnia almost every day versus never, OR = 11.74; 95% CI = [5.41, 25.49]) and smoking (smoking versus no smoking; OR = 0.60, 95% CI = [0.38, 0.92]), which indicated that the effects of lifestyle on EE and DP are slightly different.

## Discussion

In this study based on nationally representative survey data, we found suboptimal lifestyle scores, low perceived-competency, and high burnout prevalence among radiology residents in China. Furthermore, this study revealed that a healthier lifestyle was associated with higher competency, particularly in the realms of sleep, physical activity, and alcohol consumption. Meanwhile, smoking, sleep and physical activity were also the most important lifestyle factors associated with burnout.

### Sleep

Consistent with previous studies, radiology residents having adequate sleep demonstrate higher competency and lower occupational burnout in our analyses. Healthcare professionals, including radiologists, often suffer from sleep deprivation and most of them reported that they were not receiving the amount of sleep needed (23, 24). This is particularly true for residents due to their demanding work schedules, including numerous shifts and on-call responsibilities. Our results align with previous studies suggesting that adequate sleep was one supporting factor of the high perceived-competency of radiology residents, notably in systems-based practice, practice-based learning and improvement, professionalism, and interpersonal and communication skills (25–27). It is probable that sleep deprivation affects the residents’ cognitive function (28, 29), such as visual and perceptual expertise required in substantial report interpretation of daily work in radiology, thereby leading to attentional failure (30). It also has been reported that higher emotional exhaustion and depersonalization are related to sleep quality directly through insomnia, or indirectly through addictive behaviors such as bedtime smartphone use (31, 32). Complicated and critical working environment and high level of workforce stress can easily induce negative psychological (33), while sleep deprivation or circadian disorders may partly aggravate feelings of burnout and the chronic depletion of energy stores (34, 35). A study conducted in medical students in Hong Kong had shown that high EE was predicted by sleep quality, also high DP was predicted by sleep quality (36). Moreover, in the current studies, sleeping habits were associated with all subscales of burnout, which was consistent with our findings (36).

### Physical activities

Similarly, radiology residents engaging in regular physical activities experienced lower risk of occupational burnout and better competency-based performance in our analyses, which is consistent with previous studies (7, 37–39). Physical activity, enhancing an improved sense of self-control and greater social interaction, has been confirmed of antidepressant and anxiolytic effects and protecting against harmful consequences of stress, which may have positive implications for mental health (40) and decrease risk of burnout, especially emotional exhaustion (41). Moreover, radiology residents spend most of their time working in a seated position in daily work, taking an average of only 294 steps per hour (12). Such sedentary behavior increases the risk of musculoskeletal discomfort (42) and eyestrain (43), causing metabolic disorders (44), and weakening the benefit of exercise on health and fatigue relief (45, 46). Conversely, the immense stress of residents due to heavy workload could be alleviated by regular physical activities (47).

### Alcohol and smoke

Interestingly, residents with high alcohol consumption reported higher competency than those who consumed little alcohol, and smokers reported lower risk of burnout than non-smokers. Although this may seem counterintuitive, several potential explanations exist for these findings. Firstly, in Chinese culture, social events often involve drinking, potentially enhancing interpersonal and communication skills (48, 49). Alternatively, alcohol consumption and smoking could be a compensatory behavior to cope with stress and depression; when the residents have no choice but to compromise their personal health for better psychology and better performance at work, they might turn to these substances for immediate relief (50). Furthermore, previous study has also highlighted that stress increases cigarette cravings (51), suggesting that satisfying these desire may momentarily alleviate stress and, to some extent, decrease burnout risk. Burnout often leads to a negative approach to work and poor lifestyle choices including smoking (52). Additionally, it was also reported that smoking was associated with burnout (53).

Chinese standardized residency training aims to improve residents’ core competencies (54). It is equally essential to pay attention to occupational psychology to sustain radiologists’ performance. Residents, burdened with responding to patients and senior medical staff, suffer from circadian clock disruptions and exhaustion. Moreover, increased screening programs and related responsibilities have intensified the workload for radiologists, making a healthy lifestyle more challenging. Still, the residents had a higher rate of attentional failures during prolonged work hours and on-call shifts (30, 55), leading to energy depletion, decreased interest and further deterioration of their lifestyles, which are the maladaptation to their busy schedules and stress coping mechanisms (53), and further affect residents’ perceived-competency and increasing burnout risk, potentially reinforcing a vicious cycle (56). Therefore, it is necessary and urgent to focus on the modification of lifestyle behaviors among radiology residents, especially their sleep, smoking, physical activities and drinking habits. Reasonable strategies could include incorporating non-exercise activity thermogenesis (NEAT) into work routines—ensuring adequate hydration, taking regular breaks, performing basic exercises, and monitoring caloric intake (23, 44)—and scheduling night shifts to allow for sufficient sleep. It is not ethical nor sustainable for residents to sacrifice personal health for professionalism and performance. Our findings also indicated that the changes in lifestyles among this vulnerable group may contribute to enhancement in competency. In addition, systemic/organizational interventions are needed as well as individual management. Human factors and ergonomics (HFE) in healthcare delivery is helpful to optimize clinician wellbeing and clinical outcomes (57). Previous studies have shown that the system performance and safety will be improved by reducing distractions in workplace, operating the standard procedure for patient identification (58), and taking the organization level safety workshops (59).

## Strengths

To the best of our knowledge, this was the first national representative study conducted among radiology residents in 31 provinces across China. Also, as radiology residents in standardized residency training have substantial potential for improvement in various areas such as patient care, medical knowledge, and professionalism, it is critical to focus on enhancing their performance. Our study focused on competence and psychological state, offering a comprehensive examination of the relationship between lifestyle behaviors and competency-based performance, including four lifestyle components and six competency scales; moreover, the relationship between lifestyle behaviors and burnout was studied firstly in radiology residents in China. Our findings pointed to a set of practical and modifiable lifestyle factors in such direction.

## Limitations

Nevertheless, some limitations need to be acknowledged. First, our findings were based on a cross-sectional study that measures self-perceived competence, self-reported lifestyle factors, and burnout, which were not intended to establish causal relationships. Nonetheless, the lifestyles and overall competency (e.g., compassion, communication skills, etc.) of residents are not paid as much attention compared to the completion rates of reports and assessment results. Our questionnaire specifically solicited respondents’ “current” perceived competence (as of now) and their “usual” lifestyle (retrospective, long-term measures), offering valuable insights into how individuals perceive their capabilities and manage their professional lives. This preliminary data can serve as a foundation for future research aimed at exploring causal relationships. Meanwhile, understanding these self-assessments is crucial for optimizing training programs and supporting professionals’ wellbeing and effectiveness in clinical practice. Second, the average competency score of the participating residents was low [3.16 out of 9 according to the Accreditation Council for Graduate Medical Education (17)], limiting the range of the outcome and thus the robustness of the model estimation. This may be associated with differences in medical education between China and the United States. Over 90% of residents in our study had just completed their bachelor’s degree, whereas in the US, residents qualified to participate in ACGME-accredited residency programs typically have completed both a four-year undergraduate program and four-year doctoral education. Third, the study was not able to fully elucidate the link between occupational burnout and competency-based performance. More nuanced analyses should be followed on work stressors and burnout that may adversely affect competency-based performance. For example, studies have shown that life satisfaction and burnout could influence job performance through work engagement (60). We acknowledge these limitations but believe our study have separated the participants by levels of healthfulness and demonstrated their meaningful differences.

## Conclusion

In conclusion, using a large nationwide sample in China, we demonstrated an association between lifestyle behaviors and both competency-based performance and occupational burnout. Notably, improving the quality of lifestyles, such as reducing sedentary behaviors, increasing physical activities and managing sleep, could contribute to higher competency and lower risk of burnout, especially in residents facing demanding work schedules with long daily hours and frequent night shifts. Policymakers and hospital administrators should prioritize the promotion of residents’ wellbeing, particularly given the risk that residents may turn to increased alcohol consumption and smoking as coping mechanisms for stress and fatigue.

## Data Availability

The raw data supporting the conclusions of this article will be made available by the authors, without undue reservation.
