# Supplementary material for: The price of pressure: nationwide survey on lifestyle disturbances, occupational burnout and compromised perceived-competency among radiology residents in China
Source: Front Public Health. 2024 Oct 23;12:1472397. doi: 10.3389/fpubh.2024.1472397 (PMC11538024; doi:10.3389/fpubh.2024.1472397)
Supplement: Supplementary file 1 [file Table_1.docx]

**Supplementary Table 1**

Spearman’s correlation of sleep, physical activity, smoke, and alcohol assumption.

|  | Sleep | Physical activity | Smoke | Alcohol Assumption |
| --- | --- | --- | --- | --- |
| Sleep | 1.000 |  |  |  |
| Physical activity | 0.101^*^ | 1.000 |  |  |
| Smoke | 0.062^*^ | 0.019 | 1.000 |  |
| Alcohol Assumption | 0.061^*^ | -0.050^*^ | 0.197^*^ | 1.000 |

**Supplementary Table 2**

Differences and 95% CI of perceived-competency according to continuous change in lifestyle score of radiology residents.

| Variables | | Patient care | | Medical knowledge | | Systems-based practice | | Practice-based learning and improvement | | Professionalism | | Interpersonal and communication skills | | Overall | |
| --- | --- | --- | --- | --- | --- | --- | --- | --- | --- | --- | --- | --- | --- | --- | --- |
|  |  | *β* | 95% CI | *β* | 95% CI | *β* | 95% CI | *β* | 95% CI | *β* | 95% CI | *β* | 95% CI | *β* | 95% CI |
| Overall healthy lifestyle | |  |  |  |  |  |  |  |  |  |  |  |  |  |  |
|  | Unadjusted^1^ | **-0.14** | **(-0.24, -0.04)** | -0.05 | (-0.16, 0.07) | -0.03 | (-0.14, 0.09) | 0.03 | (-0.09, 0.15) | 0.10 | (-0.02, 0.23) | 0.08 | (-0.06, 0.22) | 0.00 | (-0.10, 0.10) |
|  | Adjusted^2^ | 0.08 | (-0.02, 0.17) | **0.14** | **(0.03, 0.26)** | **0.19** | **(0.07, 0.30)** | **0.22** | **(0.10, 0.35)** | **0.25** | **(0.12, 0.38)** | **0.24** | **(0.10, 0.39)** | **0.19** | **(0.08, 0.29)** |
| Sleep | |  |  |  |  |  |  |  |  |  |  |  |  |  |  |
|  | Unadjusted | -0.12 | (-0.30, 0.06) | 0.00 | (-0.20, 0.20) | 0.07 | (-0.14, 0.28) | 0.12 | (-0.10, 0.34) | 0.21 | (-0.02, 0.44) | **0.27** | **(0.02, 0.53)** | 0.09 | (-0.09, 0.28) |
|  | Adjusted | 0.10 | (-0.07, 0.28) | **0.23** | **(0.04, 0.43)** | **0.31** | **(0.11, 0.52)** | **0.33** | **(0.11, 0.55)** | **0.40** | **(0.17, 0.63)** | **0.49** | **(0.24, 0.75)** | **0.31** | **(0.13, 0.49)** |
| Physical activity | |  |  |  |  |  |  |  |  |  |  |  |  |  |  |
|  | Unadjusted | **0.43** | **(0.22, 0.65)** | **0.44** | **(0.20, 0.68)** | **0.58** | **(0.33, 0.83)** | **0.63** | **(0.36, 0.89)** | **0.76** | **(0.48, 1.03)** | **0.68** | **(0.37, 0.99)** | **0.59** | **(0.36, 0.81)** |
|  | Adjusted | **0.48** | **(0.28, 0.69)** | **0.59** | **(0.35, 0.82)** | **0.69** | **(0.45, 0.94)** | **0.71** | **(0.45, 0.97)** | **0.90** | **(0.62, 1.18)** | **0.82** | **(0.52, 1.13)** | **0.70** | **(0.48, 0.92)** |
| Smoking | |  |  |  |  |  |  |  |  |  |  |  |  |  |  |
|  | Unadjusted | **-0.51** | **(-0.75, -0.28)** | **-0.33** | **(-0.60, -0.06)** | **-0.43** | **(-0.71, -0.16)** | -0.25 | (-0.54, 0.04) | -0.27 | (-0.58, 0.03) | -0.32 | (-0.66, 0.02) | **-0.35** | **(-0.60, -0.11)** |
|  | Adjusted | -0.19 | (-0.42, 0.05) | -0.10 | (-0.36, 0.16) | -0.15 | (-0.42, 0.12) | 0.02 | (-0.28, 0.31) | -0.14 | (-0.45, 0.17) | -0.16 | (-0.51, 0.18) | -0.12 | (-0.36, 0.12) |
| Alcohol consumption | |  |  |  |  |  |  |  |  |  |  |  |  |  |  |
|  | Unadjusted | **-0.90** | **(-1.19, -0.62)** | **-0.71** | **(-1.04, -0.38)** | **-0.81** | **(-1.15, -0.47)** | **-0.83** | **(-1.19, -0.48)** | **-0.63** | **(-1.00, -0.25)** | **-0.79** | **(-1.20, -0.37)** | **-0.78** | **(-1.08, -0.48)** |
|  | Adjusted | **-0.32** | **(-0.63, -0.01)** | **-0.44** | **(-0.80, -0.09)** | **-0.41** | **(-0.78, -0.05)** | **-0.46** | **(-0.86, -0.07)** | **-0.57** | **(-0.98, -0.15)** | **-0.72** | **(-1.18, -0.25)** | **-0.49** | **(-0.81, -0.16)** |

^1^ unadjusted: crude association

^2^ multivariable model adjusted for gender (male, female), age (≤25 years, 25 years < x ≤30 years, >30 years), region (east, central region, west, north east), degree (bachelor, master, doctoral), marital status (married, unmarried), children (yes, no), work years ($\leq$3 years, $>$3 years), work hours per day ($\leq$8 hours, $>$8 hours), number of night shifts ($\leq$1 time/month, 2-3 times/month, 1-2 times/week, $\geq$3 times/week), residency training year (1st year, 2nd year, 3rd year) and residency training site (general tertiary A, specialist tertiary A, other)

**Supplementary Table 3**

Subgroup analyses of the relationship of lifestyle and perceived-competency by gender, age, region, degree, marital status, children, work years, work hours per day, number of night shifts, residency training year and residency training site.

|  | Healthy lifestyle score | | |
| --- | --- | --- | --- |
|  | low (0-2.5] | medium (2.5-3] | high (3-4] |
| Mean competency | 0.01 (-0.23,0.26) | 0.54 (0.00, 1.07) | 1.05 (0.42, 1.69) |
|  |  |  |  |
| Total population | Ref | 1.08 (0.91, 1.30) | 1.21 (0.98, 1.50) |
|  |  |  |  |
| Male | Ref | 0.97 (0.75, 1.24) | 1.05 (0.76, 1.47) |
| Female | Ref | 1.23 (0.95, 1.58) | 1.39 (1.04, 1.86) |
| p-difference | 0.166 | | |
|  |  |  |  |
| ≤25 years | Ref | 0.87 (0.56, 1.35) | 0.92 (0.55, 1.55) |
| 25 < x ≤30 years | Ref | 1.14 (0.92, 1.41) | 1.31 (1.02, 1.70) |
| >30 years | Ref | 1.02 (0.63, 1.63) | 1.06 (0.58, 1.97) |
| p-difference | 0.481 | | |
|  |  |  |  |
| East | Ref | 0.97 (0.73, 1.29) | 1.33 (0.95, 1.86) |
| Central region | Ref | 1.03 (0.69, 1.54) | 1.07 (0.67, 1.72) |
| West | Ref | 1.19 (0.88, 1.61) | 1.26 (0.86, 1.84) |
| North East | Ref | 0.99 (0.33, 2.27) | 0.55 (0.21, 1.48) |
| p-difference | 0.715 | | |
|  |  |  |  |
| Bachelor | Ref | 1.11 (0.92, 1.33) | 1.18 (0.94, 1.49) |
| Master | Ref | 0.97 (0.47, 2.00) | 1.59 (0.72, 3.51) |
| Doctoral | Ref | 0.47 (0.10, 2.10) | 0.82 (0.16, 4.13) |
| p-difference | 0.522 | | |
|  |  |  |  |
| Married | Ref | 1.26 (0.90, 1.77) | 1.11 (0.72, 1.70) |
| Unmarried | Ref | 1.03 (0.84, 1.27) | 1.24 (0.96, 1.59) |
| p-difference | 0.830 | | |
|  |  |  |  |
| No children | Ref | 1.04 (0.86, 1.26) | 1.18 (0.94, 1.49) |
| Have children | Ref | 1.35 (0.87, 2.11) | 1.39 (0.76, 2.52) |
| p-difference | 0.368 | | |
|  |  |  |  |
| Short years | Ref | 1.15 (0.93, 1.41) | 1.31 (1.02, 1.68) |
| Long years | Ref | 0.96 (0.69, 1.33) | 0.96 (0.61, 1.50) |
| p-difference | 0.191 | | |
|  |  |  |  |
| Short hours per day | Ref | 0.87 (0.70, 1.08) | 1.00 (0.78, 1.29) |
| Long hours per day | Ref | 1.64 (1.19, 2.25) | 1.76 (1.15, 2.70) |
| p-difference | **0.003** | | |
|  |  |  |  |
| Once a month or none | Ref | 0.90 (0.71, 1.15) | 1.04 (0.78, 1.38) |
| 2-3 times a month | Ref | 1.07 (0.72, 1.59) | 1.25 (0.74, 2.12) |
| 1-2 times every week | Ref | 1.42 (0.96, 2.10) | 1.49 (0.90, 2.45) |
| ≥3 times every week | Ref | 1.98 (0.84, 4.69) | 2.17 (0.73, 6.45) |
| p-difference | **0.011** | | |
|  |  |  |  |
| First year | Ref | 0.85 (0.59, 1.22) | 1.07 (0.71, 1.60) |
| Second year | Ref | 1.01 (0.74, 1.37) | 1.05 (0.71, 1.55) |
| Third year | Ref | 1.29 (0.98, 1.70) | 1.44 (1.02, 2.02) |
| p-difference | 0.183 | | |
|  |  |  |  |
| General tertiary A | Ref | 1.09 (0.91, 1.31) | 1.25 (1.01, 1.56) |
| Specialist tertiary A | Ref | 0.63 (0.14, 2.79) | 0.37 (0.06, 2.11) |
| Other | Ref | 0.20 (0.01, 3.61) | 0.04 (0.00, 2.66) |
| p-difference | 0.303 | | |

**Supplementary Table 4**

Differences of lifestyle behaviors in work hours per day and numbers of night shifts.

|  | | **Work hours per day** | | |  | **Numbers of night shifts** | | | | |
| --- | --- | --- | --- | --- | --- | --- | --- | --- | --- | --- |
|  |  | Short hours | Long hours | *p*-value |  | Once a month or none | 2-3 times a month | 1-2 times every week | ≥3 times every week | *p*-value |
| **Sleep** | Insomnia | 61.26% | 38.74% | **<0.001** |  | 54.47% | 18.83% | 20.19% | 6.50% | **<0.001** |
|  | Basically normal | 72.86% | 27.14% |  |  | 59.73% | 18.48% | 18.29% | 3.50% |  |
|  | Normal | 78.67% | 21.33% |  |  | 67.48% | 14.49% | 14.86% | 3.17% |  |
| **Physical activity** | Irregular | 70.50% | 29.50% | **<0.001** |  | 60.34% | 17.67% | 17.57% | 4.43% | **0.009** |
|  | Regular | 78.29% | 21.71% |  |  | 66.54% | 13.70% | 16.41% | 3.36% |  |
| **Smoke** | Yes | 71.33% | 28.67% | 0.820 |  | 58.00% | 12.00% | 24.67% | 5.33% | **0.049** |
|  | No | 72.18% | 27.82% |  |  | 61.80% | 17.04% | 17.01% | 4.15% |  |
| **Alcohol consumption** | High | 71.20% | 28.80% | 0.878 |  | 54.80% | 17.20% | 22.40% | 5.60% | **<0.001** |
|  | Moderate | 71.93% | 28.07% |  |  | 59.27% | 17.37% | 19.47% | 3.89% |  |
|  | Low | 72.51% | 27.49% |  |  | 65.07% | 16.22% | 14.40% | 4.31% |  |

**Supplementary Table 5**

Logistic regression model of EE and their lifestyle factors.

| **Emotional Exhaustion** | | **Low** | **Moderate** | | | **High** | | |
| --- | --- | --- | --- | --- | --- | --- | --- | --- |
|  |  |  | **OR** | **95%CI** | ***p*-value** | **OR** | **95%CI** | ***p*-value** |
| **Smoking** |  | Reference |  |  |  |  |  |  |
|  | No(ref) |  |  |  |  |  |  |  |
|  | Yes |  | 0.68 | (0.42, 1.11) | 0.121 | 0.75 | (0.47, 1.21) | 0.237 |
| **Alcohol consumption** |  | Reference |  |  |  |  |  |  |
|  | Never(ref) |  |  |  |  |  |  |  |
|  | ≤once a month |  | 1.09 | (0.89, 1.33) | 0.426 | 1.05 | (0.84, 1.32) | 0.669 |
|  | 2-4 times a month |  | 1.05 | (0.69, 1.60) | 0.815 | 1.12 | (0.73, 1.72) | 0.594 |
|  | 2-3 times a week |  | 2.30 | (0.85, 5.85) | 0.103 | 1.54 | (0.53, 4.45) | 0.423 |
|  | ≥4 times a week |  | 1.92 | (0.15, 24.99) | 0.620 | 2.31 | (0.17, 30.70) | 0.526 |
| **Sleep** |  | Reference |  |  |  |  |  |  |
|  | Normal(ref) |  |  |  |  |  |  |  |
|  | Insomnia＜ twice a month |  | 1.82 | (1.47, 2.26) | ＜0.001 | **1.98** | **(1.52, 2.56)** | **＜0.001** |
|  | Insomnia 1-2 times a week |  | 2.75 | (2.16, 3.52) | ＜0.001 | **4.37** | **(3.34, 5.72)** | **＜0.001** |
|  | Insomnia 3-5 times a week |  | 3.04 | (2.16, 4.29) | ＜0.001 | **8.87** | **(6.42, 12.26)** | **＜0.001** |
|  | Insomnia almost every day |  | 3.64 | (1.50, 8.87) | 0.004 | **15.08** | **(7.16, 31.73)** | **＜0.001** |
| **Physical activity** |  | Reference |  |  |  |  |  |  |
|  | Almost every day(ref) |  |  |  |  |  |  |  |
|  | 3-5 times a week |  | 1.28 | (0.34, 4.91) | 0.714 | 7.02 | (0.82, 59.82) | 0.075 |
|  | 1-2 times a week |  | 1.36 | (0.38, 4.87) | 0.646 | 3.90 | (0.47, 32.44) | 0.208 |
|  | ＜ twice a month |  | 1.78 | (0.50, 6.29) | 0.365 | 5.16 | (0.63, 42.34) | 0.127 |
|  | Never |  | 2.81 | (0.79, 9.96) | 0.097 | **10.06** | **(1.23, 82.51)** | **0.032** |

**Supplementary Table 6**

Logistic regression model of DP and their lifestyle factors.

| **Depersonalization** | | **Low** | **Moderate** | | | **High** | | |
| --- | --- | --- | --- | --- | --- | --- | --- | --- |
|  |  |  | **OR** | **95%CI** | ***p*-value** | **OR** | **95%CI** | ***p*-value** |
| **Smoking** |  | Reference |  |  |  |  |  |  |
|  | No(ref) |  |  |  |  |  |  |  |
|  | Yes |  | 0.756 | (0.48, 1.20) | 0.235 | **0.60** | **(0.38, 0.92)** | **0.021** |
| **Alcohol consumption** |  | Reference |  |  |  |  |  |  |
|  | Never(ref) |  |  |  |  |  |  |  |
|  | ≤once a month |  | 1.28 | (1.06, 1.54) | 0.009 | 1.26 | (1.03, 1.54) | 0.026 |
|  | 2-4 times a month |  | 1.13 | (0.76, 1.69) | 0.523 | 1.21 | (0.82, 1.77) | 0.338 |
|  | 2-3 times a week |  | 2.03 | (0.74, 5.56) | 0.165 | 1.77 | (0.67, 4.66) | 0.249 |
|  | ≥4 times a week |  | 5.17 | (0.44, 60.36) | 0.178 | 3.49 | (0.23, 52.90) | 0.368 |
| **Sleep** |  | Reference |  |  |  |  |  |  |
|  | Normal(ref) |  |  |  |  |  |  |  |
|  | Insomnia＜ twice a month |  | 1.84 | (1.56, 2.23) | ＜0.001 | 1.92 | (1.54, 2.39) | ＜0.001 |
|  | Insomnia 1-2 times a week |  | 2.34 | (1.86, 2.96) | ＜0.001 | 3.64 | (2.86, 4.63) | ＜0.001 |
|  | Insomnia 3-5 times a week |  | 2.62 | (1.86, 3.68) | ＜0.001 | 7.16 | (5.26, 9.76) | ＜0.001 |
|  | Insomnia almost every day |  | 3.53 | (1.40, 8.89) | 0.008 | **11.74** | **(5.41, 25.49)** | **＜0.001** |
| **Physical activity** |  | Reference |  |  |  |  |  |  |
|  | Almost every day(ref) |  |  |  |  |  |  |  |
|  | 3-5 times a week |  | 0.68 | (0.25, 1.82) | 0.439 | 3.41 | (0.69, 16.90) | 0.134 |
|  | 1-2 times a week |  | 0.60 | (0.23, 1.52) | 0.280 | 2.63 | (0.55, 12.62) | 0.227 |
|  | ＜ twice a month |  | 0.65 | (0.26, 1.65) | 0.368 | 2.93 | (0.62, 13.90) | 0.177 |
|  | Never |  | 0.77 | (0.31, 1.94) | 0.582 | 4.55 | (0.96, 21.60) | 0.057 |
